# Frameworks to analyse accessibility of public recreational spaces for persons with disabilities: a rapid scoping review

**DOI:** 10.1080/16549716.2026.2729891

**Published:** 2026-09-14

**Authors:** A. Hana Miller, Sagar Prasai, Joanna Morrison

**Affiliations:** a Department of Medicine, University of British Columbia, Vancouver, Canada; b Diverse Patterns, Kathmandu, Nepal; c Institute for Global Health, University College London, London, UK

**Keywords:** Disabilities, accessibility, recreation, equity, physical activity

## Abstract

The global population currently struggles to meet the physical activity guidelines. This is exacerbated for persons with disabilities (PWD). There is increasing acknowledgement of the need for recreational public spaces to be accessible and provide opportunities for physical activity. Designing and evaluating facilities requires tools and frameworks that capture diversity and consider access beyond the physical environment. Through a rapid scoping review, we sought to describe peer-reviewed literature that developed or implemented frameworks (conceptual or analytical) to analyse access for PWD in public recreational spaces. A comprehensive literature search on Web of Science was conducted. Studies were included if published in English between 2005 and 2025 and developed or implemented a framework to assess the inclusivity of public recreational spaces for PWD. Titles, abstracts, and full texts were independently screened by two reviewers. Reference lists of included papers were searched. Relevant papers were extracted. Of 872 identified papers, 42 underwent full-text review, and 20 (16 from the original search and 4 from reference lists) were included. All were based in middle- and high-income countries. Most used an established framework rather than developing their own. Frameworks were categorised as conceptual or analytical, with most being analytical. Inclusion of PWD in framework use or design was not consistently stated. All but one framework focused on the needs of people with physical disabilities. Ensuring access to public parks for persons with disabilities is an overlooked strategy to increasing recreational physical activity, particularly in low-income countries. Current frameworks need testing and adaptation for these contexts.

## Paper Context

- **Main findings:** Our global review found and described 20 published frameworks that were used in high and middle-income countries to evaluate access to public recreational spaces for persons with physical and visual impairments.
- **Added knowledge:** This review highlights the need for evaluation frameworks to understand the accessibility of recreational space in low-income countries for persons with a diverse range of disabilities, and explicit inclusion of persons with disabilities in the framework development and implementation process.
- **Global health impact for policy and action:** Addressing inequities in park access to align with the WHO Global Action Plan on Physical Activity 2018–2030 can guide more equitable policies and interventions that increase physical activity participation among marginalized populations, including people with disabilities.

## Background

In 2022, nearly a third of the global adult population did not meet the recommended levels of physical activity [1]. The number of people failing to meet the recommended physical activity guidelines is projected to have increased since this study was conducted and is expected to continue to rise [1]. While the challenges to achieve the daily physical activity guidelines are a global issue, they are exacerbated for persons with disabilities (PWD). Approximately 1.5 billion people worldwide experience some form of physical, mental, sensory, or intellectual disability [2]. However, PWD are 16–62% less likely than the general population to meet physical activity recommendations, leading to a higher risk of serious health problems [2].

There is increasing acknowledgement of the need to ensure that recreational public spaces such as parks are accessible and provide opportunities for physical activity [3]. Public recreation spaces are spaces where people can be active, or socialise and gather with others [4]. The design of new public spaces and adaptation of existing spaces should reflect the diverse needs of the general population. Design and evaluation of existing facilities need tools and frameworks that are able to capture this diversity and consider access beyond the physical environment [5]. The WHO’s International Classification of Functioning, Disability and Health is a conceptual framework that provides a starting point for considering person–environment interactions and contextual barriers and facilitators to full and active participation in community life for people with disabilities [6,7]. Tools to evaluate environmental factors are non-specific and are not easily translatable to the public park environment [8]. The Universal Design conceptual framework – a framework that promotes the design of products and the environment to be accessible for all people without the need for adaptation [9] – has been used across disciplines. Under the United Nations Convention on the Rights of Persons with Disabilities (CRPD), Universal Design is recognized as a core principle that moves beyond separate ‘special accommodations’ by promoting approaches that make spaces, services, and products usable for the widest range of people from the outset, while acknowledging that some individuals may still require supportive or adaptive technologies [10]. Universal design, however, is challenging to operationalise [11]. Therefore, through a rapid scoping review, we sought to describe peer-reviewed literature, which has developed or implemented frameworks (conceptual or analytical) to analyse access for PWD in public recreational spaces.

### The health benefits of parks

Studies have found that access to green spaces, such as parks, is associated with reduced feelings of stress, anxiety, and depression [12]. Possible mechanisms that have been identified include better air quality, increased physical activity, connection with nature, and social contact [13–15]. The positive impact of parks is applicable across the lifespan, spanning from young children to older adults [16]. While various causal pathways have been proposed to describe the relationship between access to parks and improved mental wellbeing, Barton and Rogerso (2017) proposed that parks can promote both acute positive psychological experiences and physical activity, which, in turn, can promote well-being [17]. Physical activity has long been acknowledged as a key component of good mental and physical health. Physical activity can reduce the risk of non-communicable diseases, such as cardiovascular diseases and type 2 diabetes, in a dose-dependent relationship [18]. Physical activity also has positive effects on mood and quality of life. More specifically, physical activity has been shown to aid with sleep and improve various psychiatric disorders [19].

The World Health Organization (WHO) recommends that adults engage in at least 150 minutes of moderate-intensity physical activity weekly, at least 75 minutes of vigorous-intensity physical activity, or a combination of both [20]. Despite these recommendations and the clear benefits of physical activity, studies have estimated that approximately 3.2 million deaths per year can be attributed to insufficient levels of physical activity [21]. This number may continue to rise as countries develop economically. The WHO has stated that increases in sedentary behaviour are due to factors such as changing transport patterns, increased use of technology, cultural values, and behaviour [20]. Interventions, across the individual, group, and community context [22], are needed to address the decreasing engagement in physical activity.

### Importance of frameworks

To ensure that everyone has equal access to the health benefits of public parks, it is important to analyse the extent to which they meet the needs of their local population. When access is not equal, it is important to develop and implement ways to mitigate this. Access is a complex issue to evaluate, as it should encompass biological, psychological, and social dimensions in order to provide a comprehensive understanding of the person–environment interaction [23]. To evaluate the accessibility of public parks for PWD, frameworks can be a helpful tool. Frameworks can be dichotomized into analytical and conceptual frameworks, both of which can help designers and policy makers ensure that they are capturing the multi-dimensional aspects of access. Analytical frameworks provide guidance of how and what to measure and evaluate [24], and conceptual frameworks describe relationships between concepts related to accessibility [25]. Both types of frameworks can aid in interpreting evaluation results and developing theories about how park adaptations or interventions can improve access, while also helping to determine whether the same interventions or adaptations may be effective in other contexts.

## Methods

**Figure 1.**
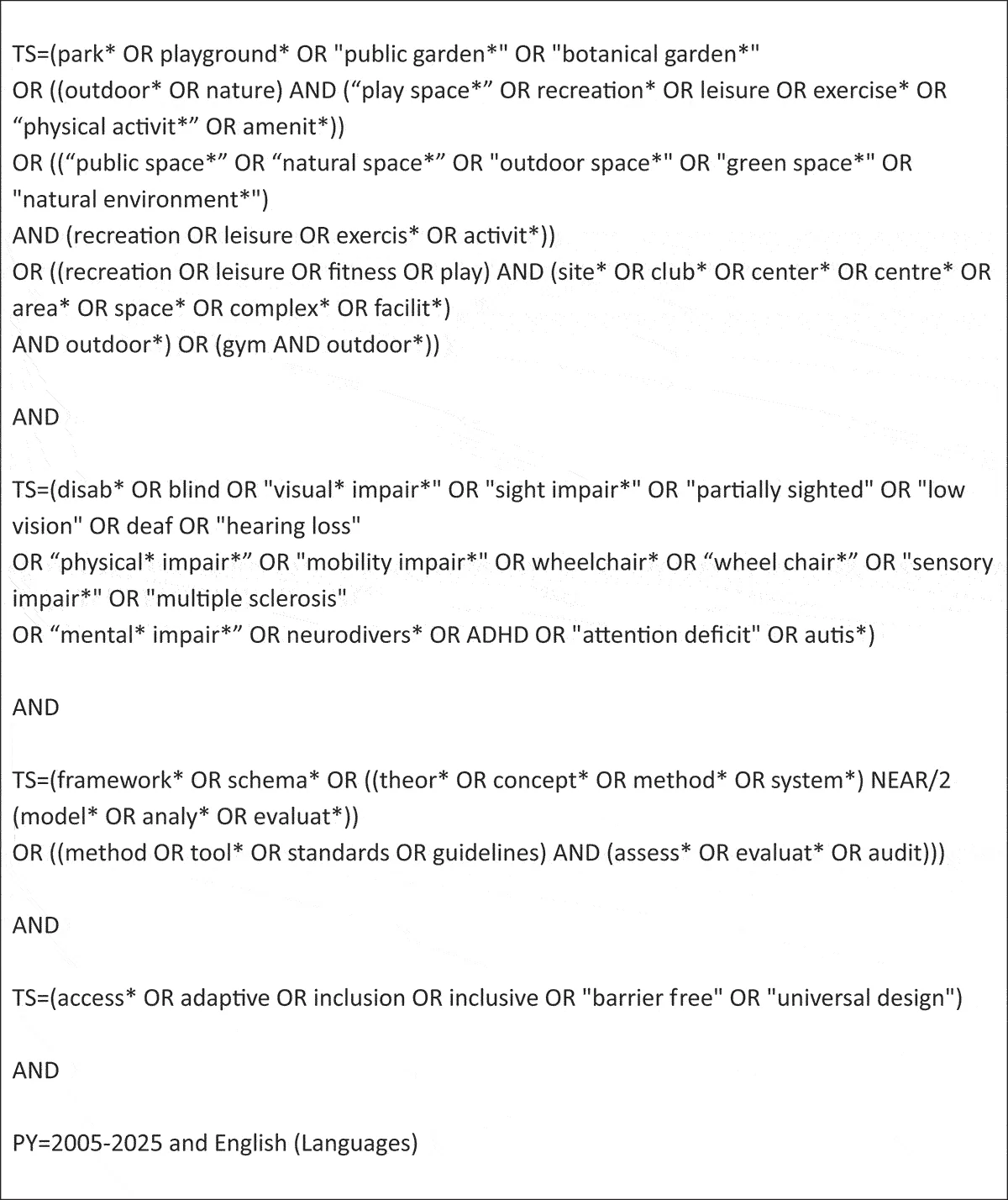
Complete search strategy.

This review was a rapid scoping review. A rapid review is characterized by a synthesis of information in a systematic and resource-efficient manner [26]. Although the review protocol was not registered, a structured protocol that was developed a priori (Appendix A) was followed. The review was conducted to inform the development of an evaluation framework as part of ongoing research (not reported here – reference removed for anonymisation) which made it time sensitive. There was deviation from our protocol in one aspect. Planned searches in Scopus, Web of Science, ASSIA, Jstor, Embase, Google Scholar, and Google, and in relevant international organisations (United Nations Children’s Fund, International Development Association, Humanity and Inclusion, CBM Global Disability and Inclusion, United Nations Human Rights Agency) were not conducted. An initial search revealed a pre-screening volume of literature that was beyond the capacity of the team to screen within the allocated time of HM’s studentship. Therefore, only one database, Web of Science, was searched on 26 March 2025. Search terms covered the key concepts of recreation space, disability, framework, and accessibility. The complete search strategy is listed in Figure 1.

For the purposes of this review, the key terms were defined as follows:

Recreation space: settings for leisure and physical activity [4].

Disability: any physical, sensory, mental, and intellectual and developmental impairment or disability mentioned as such by authors [27].

Framework: A framework refers to a structure, overview, outline, system, or plan that consists of a variety of descriptive categories and the relations between them [28].

Accessibility: Accessibility describes a state in which an individual’s functional capabilities and the functional demands of the environment are aligned [29].

### Study selection

To be included, studies needed to develop or implement a framework to assess the inclusivity of public recreational spaces for PWD. Studies that developed or implemented frameworks with a focus solely on children were excluded. To manage the search within a short time frame, we limited our search to articles between 1 January 2005 and 26 March 2025. Reviewers were only able to read English, and therefore we only included articles published in English. While there were no limitations on what country the study was conducted in, we recognize the capacity to only include English articles may indirectly limit the countries included in this review.

### Search strategy

Titles and abstracts, followed by full texts, were independently screened by two reviewers. Conflicts were resolved through discussion. The reference list of included papers was searched for relevant papers that may have been missed by the original search. The data extraction form was developed in Microsoft Excel by HM. JM and HM independently extracted data from two papers, compared and then adjusted the extraction sheet after discussion. Thereafter, extraction was done independently, half by HM and the other half by JM. Data extraction was driven by the research question – study context, study characteristics, and framework characteristics. Framework characteristics included categories such as key components of the framework, method of measurement for each component, whether any standards were used (e.g. Americans with Disabilities Act [30]), and whether PWD participated in developing or implementing the framework [31,32].

### Data synthesis

The synthesis of the papers follows a narrative synthesis approach. Narrative synthesis enables the analysis of similarities and differences between studies, summary of knowledge, and exploration of relationships within the data [33]. After extraction and synthesis, the authors consulted with two individuals with disabilities in Nepal, seeking their perspective on the search and the findings [31,32].

## Results

**Figure 2.**
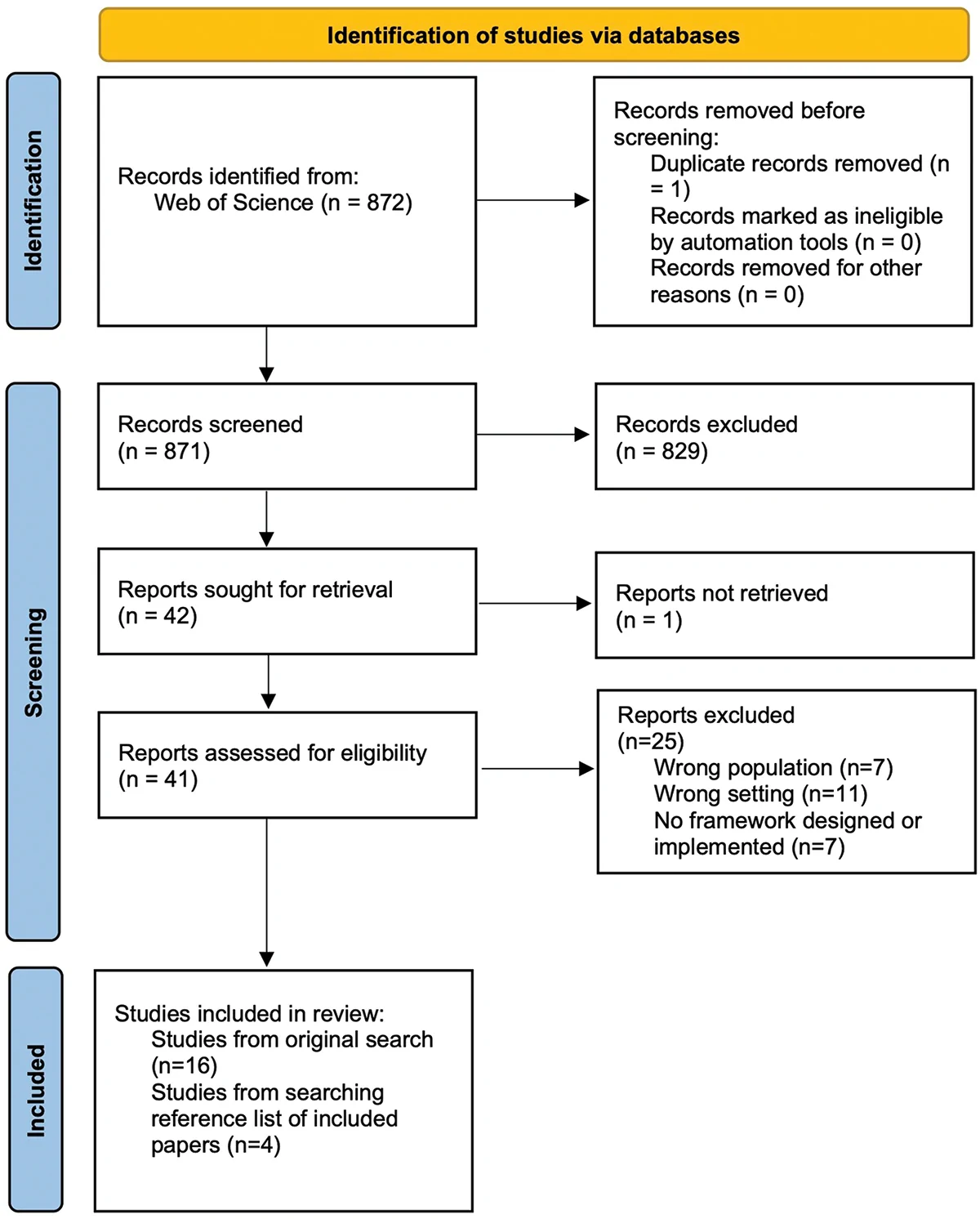
Preferred reporting items for systematic review and meta-analysis (PRISMA) flow diagram [54].

Through searching Web of Science, 872 papers were initially identified. After removing duplicate papers, 871 papers were title and abstract screened. Forty‑two papers were sought for retrieval. One paper was unable to be retrieved, leaving 41 papers for full-text review. Twenty‑five papers were then excluded. The papers were excluded for either not presenting a clear framework, not being in the context of parks and outdoor recreational facilities, not designing or implementing their framework in the context of PWD, or a combination of these reasons. HM and JM independently noted the reasons for exclusion or inclusion in Rayyan, and disagreements were resolved through discussion. With borderline cases, we prioritised the development and/or implementation of a framework and considered its potential usability in relation to ongoing research. In total, 20 papers were included in the scoping review − 16 papers from the original search and 4 papers from searching their reference lists (Table 1) One of the papers (Rimmer et al [34]) which was found through searching the reference lists of included papers was outside our date range of inclusion criteria [34]. We decided to include it because it reported on the original framework that was used in a later paper (Rimmel et al 2017) that was included in our review [39]. The paper identification and selection process is outlined in Figure 2.

**Table 1.** Summary of frameworks included in the review.

Title	Author	Year	Country	Setting	Key components of the framework	Framework type (conceptual or analytical)	Reported consultation of PWD in framework development
Development and validation of AIMFREE Accessibility Instruments Measuring Fitness and Recreation Environments [34]	Rimmer et al.	2004	United States	Fitness facilities	General measures of accessibility for bathrooms, elevators, entrance areas, parking lot, telephones, water fountains. Fitness centre specific measures of accessibility include equipment, fitness programme, hot tubs/saunas, information, locker rooms, policies, professional behaviour, professional knowledge/attitudes, professional support and training, swimming pool.	Analytical	Yes
Accommodation of wheelchair-reliant individuals by community fitness facilities [35]	Dolbow and Figoni	2015	United States	Community centre	Staffing, specialised equipment, customer service desk, accessibility of equipment, drinking fountains, restrooms, elevators, travel pathway, entrance/doors, ramps and parking. Long checklist available in appendix of manuscript.	Analytical	No
Measuring Physical Activity in Outdoor Community Recreational Environments: Implications for Research, Policy, and Practice [36]	Aytur et al.	2015	United States	Outdoor community recreation environments	**Accessibility, use, usability, quality, safety, barriers, facilitators.**	Conceptual	No
Methodology for Evaluating the Usability of Public Equipment for Physical Activity: An Approach to Interface with Blind and Low Vision Individuals [37]	de Oliveira and Okimoto	2015	Brazil	Public exercise equipment	Loose framework that evaluates ergonomic, usability, familiarity, self-experience, and emotional factors.	Analytical	Yes
Evaluation of the accessibility of public green spaces [38]	Halajová	2016	Slovakia	Public green spaces	The evaluation of the accessibility of green spaces, the evaluation of barrier free paths (sidewalks), the evaluation of park furniture, and the evaluation of playgrounds.	Analytical	No
Fitness facilities still lack accessibility for people with disabilities [39]	Rimmer et al.	2017	United States	Fitness facilities	Access routes and entrance areas, parking, equipment, information and signage, locker rooms and showers, hot tubs, whirlpools, saunas, and steam rooms (spas), bathrooms, telephones, water fountains, professional support/training, policies, programs, swimming pool, elevators, and professional behavior.	Analytical	Yes
Lived Experiences and Technology in the Design of Urban Nature Parks for Accessibility [40]	Poldma et al.	2017	Canada	City park	Physical, visual and activity characteristics of the physical site and throughout the walkabouts, the obstacles and features of the site during these activities, and how way-finding occurs.	Conceptual	Yes
Accessibility and usability of parks and playgrounds [41]	Perry et al.	2018	New Zealand	Public parks and playgrounds	Access to transportation, physical characteristics of the environment (eg. width of main paths), lighting, routes to elevated components, rest areas, areas to relax, presence of restroom, and presence of a drinking fountain.	Analytical	Yes
Developing a measure of user-perceived universal design for sport facilities [42]	Kim and Chang	2018	South Korea	Public sport facilities	Equitability, usability, convenience, aesthetics, safety, pleasantness.	Analytical	No
Ten quality criteria of the public spaces in a large city [43]	Kozlova and Kozlov	2018	Russia	Public spaces	Accessibility, safety, legibility, sustainability, human scale, identity, interactivity, flexibility.	Conceptual	No
Examining Public Acceptance towards Physical Activity Involvement of People with Disabilities (PWD) – A SEM Approach [44]	Ong and Rashid	2019	Malaysia	Public recreation parks	Personality, subjective norms, and (exposure and ethnicity leading to attitude) leading to public acceptance.	Conceptual	No
Age-Friendly Features in Home and Community and the Self-Reported Health and Functional Limitation of Older Adults: the Role of Supportive Environments [45]	Choi	2020	United States	Urban communities	Through binary measurements, the framework measures the perceived importance of items in each domain, perceived availability of items in each domain, self-rated health, functional limitations, and person environment fit. The domains include housing, outdoor spaces and buildings, transportation, health and wellness, social participation and inclusion, volunteering and civic engagement, job opportunities, and community information.	Analytical	No
Analysis of Accessibility of Green Spaces in Tehran for People with Limited Movement with an Emphasis on the Concept of Universal Design [46]	Rafizadeh	2021	Iran	Parks	Safety, security, independence, and possibility of orientation.	Conceptual	No
Accessibility for Disabled People at Peshmerga Park, Erbil, Iraq [47]	Tarhuni	2022	Iraq	Public park	Parking, accessibly of main gate, pathways, ramps, toilets, playground, sports and fitness, accessibility to mosque, sitting, open theatre, and plaza.	Analytical	No
Analysing the Design Criteria of Public Open Spaces for the Disabled Persons: An Evaluation of Kumsal Park in Northern Cyprus [48]	Erçin and Hindwan	2023	Northern Cyprus	Public park	Location, accessibility, parking area, sidewalk width, sidewalk surface, lighting, signage, maintenance, security, slope, curb design, ramp materials, hardscapes, handrails, seating, parking.	Analytical	No
How Can People with Disabilities Use the Outdoors? An Assessment Within the Framework of Disability Standards [49]	Piskin and Akdeniz	2023	Turkey	Urban parks	Physical characteristics of the environment (eg. path width), characteristics of signage (eg. height of signboards), parking conditions (eg. number of disabled parking lots), seating measurements (eg. depth of seating elements).	Analytical	No
Assessment of barriers for people with disability to enjoy national parks [50]	Aguilar-Carrasco et al.	2023	Canada and Spain	National parks	Access-to-nature constraints categorized as structural, interpersonal, and intrapersonal. Structural constraints are related to the physical environment, intrapersonal constrains are related to the psychological aspects, and interpersonal constraints are related to factors that influence people’s relationships with others.	Analytical	No
Adaptive outdoor physical activities for adults with mobility disability: a scoping review [51]	Derakhshan et al.	2024	United States, Canada, Europe, Australia	Outdoor spaces	7 principals of universal design 1) equitable, 2) flexible, 3) simple and intuitive, 4) perceptible information, 5) tolerance for error, 6) low physical effort, and 7) size and space for approach and use. SEM: intrapersonal, social environmental, physical environmental, policy	Conceptual	No
Assessment of park paths and trails to promote physical accessibility among wheelchair users in Saudi Arabia [52]	Bakhsh et al.	2024	Saudi Arabia	Public parks	The framework includes specifications for accessible wheelchair seating spaces, slopes, cross slopes, and clearance spaces. The majority of factors relate to accessibility. Various components (40 items in three dimensions) are measured to help evaluate the physical accessibility of outdoor environments.	Analytical	No
Interpreting inclusive and equitable green infrastructure in the context of common prosperity: an evaluation and optimization of urban park inclusivity in Hangzhou [53]	Shi et al.	2025	China	Urban parks	support for physical capability (eg. handrails), support for sensory capability (eg. floor materials in different functional areas), and support for cognitive capability (eg. readability of guide map).	Analytical	No

### Study characteristics

The papers identified in this rapid scoping review highlighted the interdisciplinary nature of framework development and implementation in the context of access for PWD to public recreational facilities. Papers were published across a diverse range of journals, covering the following disciplines: urban development, architecture, health, disability studies, engineering, and social sciences and humanities.

The papers in this review were all based in middle- and high-income countries as classified by the World Bank [55]. There were six papers from middle-income countries, namely Turkey [49], Malaysia [44], China [53], Brazil [37], Iran [46], and Iraq [47]. The 14 high-income countries represented in this scoping review include the United States (*n* = 5) [34–36,39,45], Canada (*n* = 2) [40,50], Slovakia (*n* = 1) [38], Saudi Arabia (*n* = 1) [52], Spain (*n* = 1, combined with one of the Canadian papers) [50], Northern Cyprus (*n* = 1) [48], New Zealand (*n* = 1) [41], South Korea (*n* = 1) [42], and Russia (*n* = 1) [43]. There was one global review from high-income countries in North America, Europe, and Oceania [51].

Most papers (*n* = 11) used and implemented an established framework [35,39–41,45,47–52]. Five studies explicitly justified their selection of framework [39,40,45,48,52]. Six studies developed their own framework but did not use it [34,36,37,43,44,46], and three studies developed and implemented their own framework [38,42,53].

The inclusion of PWD to inform the use or design of frameworks was not consistently stated in most of the papers. Four papers explicitly stated their inclusion of PWD in at least one stage of their framework development or implementation [34,37,40,41]. Poldma et al. employed a constructivist approach known as the Living Lab in Canada [40]. The researchers and PWD participated in co-constructing the aspects of accessing and navigating the park in a preparatory activity and leisure activity in the park. De Oliveira and Okimoto conducted a study with blind and low‑vision users to understand their experience with outdoor fitness equipment [37]. They applied questionnaires on intuitiveness of use, satisfaction, comfort, pleasantness, accessibility, and the extent to which users were motivated to use the equipment. Perry et al. developed their framework in consultation with various groups that represented individuals with disabilities – CCS Disability Action (a disability advocacy organization in New Zealand), the Blind Foundation of New Zealand and officials of three city councils (including accessibility support groups) [41]. The framework was reviewed and modified based on consultation with these groups.

### Framework characteristics

14 frameworks were analytical [34,35,37–39,41,42,45,47–50,52,53] whereas six frameworks were conceptual [36,40,43,44,46,51]. While heterogeneity can be observed when comparing the framework characteristics (see Table 1), patterns can be noted when considering the analytical and conceptual frameworks separately.

Analytical frameworks shared common characteristics, such as being structured for data collection with a combination of quantitative and qualitative methods. The methods by which the items in the framework were quantified varied across frameworks. One framework considered the physical environment through methods such as direct measurement and rating components on a scale according to strictly defined criterion [49]. Most frameworks described the physical environment through nominal scales such as the absence or presence of certain accessible features [34,35,38,39,41–43,45,47,48,52]. One analytical framework used a combination of the two categories of measurement described above [53]. One framework used an administered questionnaire and observation of participants to measure user experience [37]. All analytical frameworks, regardless of their measurement methods, assessed features beyond the physical park equipment, including elements such as parking, signage, vegetation, and security.

In contrast to the analytical frameworks, there was greater heterogeneity observed across the conceptual frameworks. One paper provided examples of tools and measures to quantify the concepts within the conceptual framework presented, thereby allowing it to be adapted to an analytical framework. All of the conceptual frameworks included concepts that would be subjective to the individuals using the framework. For example, three of the conceptual frameworks included safety as one of the concepts in their framework [36,46,51].

While there was variability across the literature, 11 papers referenced existing accessibility standards, with the most common being the Americans with Disabilities Act (ADA) and universal design [35,37,39–42,47–49,51,53]. Four papers referenced the ADA [35,39,47,53]. The ADA is a federal civil rights law in the United States of America that protects people with disabilities in various aspects of public life, such as the opportunity for employment, purchases of goods and services, and participation in state and local government programs [30]. Four papers referenced universal design [37,40,42,51]. Universal design influences the design process with the goal of ensuring the design is useful, adaptable, understandable, effective, safe, comfortable, and spacious for diverse users [51]. While universal design was operationalized in a variety of ways, the seven core principles include equitable use, flexibility, simplicity and intuitive use, perceptible information, tolerance for error, low physical effort, and size and space for approach and use [51].

Papers rarely reported on the length of time it took to develop the framework and/or apply it in a park. One study commented on the time it took to implement the framework at each park location [52]. While the length of time for framework development and/or implementation could be inferred in studies that required participants, they were not explicitly stated.

Five studies reported on strengths or limitations of their framework (Bakhsh, Rimmer, Rimmer, Perry, Kim). Papers reporting on the AIMFREE framework were the most transparent in their discussion of strengths and limitations of the tool, noting where some of the subscales did not perform well psychometrically, and the prohibitively long implementation time, as well as high interrater reliability and a recognised need to test the structural reliability of the tool. One study recognised the need to include PWD to test the tool (Bakhsh), one study noted the need to be able to disaggregate results by respondent type (Kim), one study noted the need to add a variable regarding surface deterioration to their tool, and also noted the need for external validation before transferability could be assessed (Perry).

All of the frameworks, except for de Oliveira and Okimoto, were designed to consider accessibility for people with physical disabilities. De Oliveira and Okimoto included individuals who were blind or had low vision [37,56]. While it was not always explicit in the abstract or title of the article, we found that there were no studies that designed or implemented frameworks specifically for people with intellectual, developmental, mental, or behavioral disabilities.

## Discussion

Through this rapid scoping review, we sought to describe the literature regarding frameworks to analyse access for people with disabilities to public recreational facilities. Through our search, we found that there were no published frameworks developed or implemented in low-income countries. We also note a focus on physical disabilities and a lack of inclusion of PWD in the framework development and implementation process.

The studies included in this scoping review were conducted exclusively in middle- and high-income countries, highlighting a geographic and economic concentration in research using accessibility frameworks for recreational environments [2]. This aligns with prior findings in the literature that, despite 80% of PWD living in LMICs, there is still a scarcity of physical activity and disability data from LMICs [2]. While there were papers from middle-income countries in our review, low-income countries were not represented. While this may be due to our search strategy only including studies published in English, another hypothesized reason for this gap includes a greater focus on health equity for PWD in high-income countries [57]. Although the underlying causes are likely multifactorial, the comparatively lower emphasis on health equity for people with disabilities in LMICs is reflected in the fact that public spending on disability remains minimal in many countries [58].

Regarding framework characteristics, the review identified 14 analytical and 6 conceptual frameworks. Analytical frameworks were more standardized than conceptual frameworks. The frameworks varied with respect to their data collection techniques being quantitative, qualitative, or mixed-methods. The analytical frameworks all assessed features beyond the physical park equipment, suggesting a more holistic view of accessibility. In contrast, conceptual frameworks showed greater variability and included more subjective elements, such as perceived safety. Accessibility of parks encompasses both physical and social aspects. This aligns with the understanding of environment as set out by the International Classification of Functioning, Disability and Health (ICF), the WHO’s framework that links disability and health [59]. The ICF describes the environment as factors that range from the physical space to social attitudes, institutions, and laws [59]. Moreover, as highlighted during our consultation with individuals with disabilities, the subjective components of accessibility are vital to gaining a comprehensive understanding of accessibility. Including subjectivity into frameworks, however, can introduce bias and reduce utilisation of the framework across countries and contexts.

Perspectives of PWD were underrepresented in the papers in this scoping review. Four out of the 20 papers included PWD in at least one stage of their framework development or implementation [34,37,40,41]. It is possible that other studies included PWD in their study or authorship without explicit mention. However, despite not including PWD in the framework development or implementation, the majority of the studies developed or used frameworks exclusively aimed at PWD (*n* = 13) [34,36–40,44,46,47,49–52]. The lack of representation in the framework development or implementation reflects a broader and well-evidenced issue of PWD being significantly underrepresented in health research [60].

As referenced in the key concept definitions, disability is a broad term. However, all frameworks, except for de Oliveira and Okimoto [37], focused solely on physical disabilities, with no frameworks designed specifically for individuals with cognitive or mental health disabilities, pointing to a gap in the scope of current accessibility frameworks. Although there is significant diversity within physical disabilities, different frameworks may be more applicable to non-physical disabilities. For example, a literature review found that parents of autistic children preferred playgrounds that were smaller and quieter and preferred play activities that involved strong sensory stimuli or repetitive movements [61]. This suggests that accessibility frameworks only aimed for individuals with physical disabilities may not encapsulate critical accessibility components for individuals with other disability types.

Future research should assess the appropriateness of conceptual and analytical frameworks identified in this review to LMIC contexts. Only middle- and high-income countries were represented in this review. This is particularly important because, as countries progress through economic development and invest in public infrastructure, such as parks, applicable frameworks from similar contexts should be available to help ensure accessibility for PWD. Moreover, given the WHO’s observation that economic development can lead to decreased physical activity, the need for appropriate frameworks to evaluate park accessibility for PWDs becomes increasingly critical to promote inclusive and active environments [20]. We acknowledge that our capacity to only include English papers may have excluded some papers from non-English‑speaking low-income countries. However, among the countries included in the review, not all include English as an official language of the country. Future frameworks should also consider reporting on the length of time to implement or develop the proposed framework. This can help those seeking to evaluate or develop interventions allocate appropriate time to the task.

This review has notable limitations. This review was a rapid scoping review which was conducted to inform ongoing research and was conducted under time constraints of a studentship. The search was only conducted in one database, Web of Science, which deviated from the a priori protocol (Appendix A). On seeking advice from a librarian, this was identified to be the most appropriate database for our search. However, it is possible that more papers from low-and middle-income countries may have been discovered by expanding our search to papers not published in English and searching a more extensive range of databases, such as LILACs and African Journals online. It is common for rapid reviews to limit the number of databases searched, particularly when they are conducted primarily to inform policy or practise [26,62].

Due to the rapid nature of our review, we were unable to convene an advisory committee as recommended by Levac et al. [63] Instead, we invited SP (a person with a physical impairment) and SL (a person with a visual impairment) to comment on our search and conclusions during a structured online meeting in which the participants were presented with the scoping review’s structure, methodology, and main findings. SP and SL provided verbal consent to use their reflections to inform the qualitative interpretation of the findings. The consultation with SP and SL highlighted the need for accessibility evaluation frameworks to go beyond the largely standardized and infrastructure-focused frameworks used in many high- and middle-income countries, to include subjective and attitudinal dimensions of accessibility. Based on these findings, future evaluation frameworks should consider aligning with the WHO’s ICF, which recognizes social attitudes as environmental factors that can restrict participation.

## Conclusion

Ensuring access to public parks for PWD is an overlooked strategy to increasing recreational physical activity, particularly in low-income countries. As indicated in the WHO Global Action Plan on Physical Activity 2018–2030, differences in physical activity levels can be explained by meaningful inequities in the opportunities for physical activity [64]. Addressing these disparities in physical activity participation amongst marginalized communities, including PWD, is an underlying principle to the success of the global action plan. Using analytical and conceptual frameworks can ensure a holistic evaluation of access and can enable informed intervention planning to increase equity of access. This review identified 20 different frameworks that have been developed in middle- and high-income countries. There is a need to test their appropriateness in low-income countries and adapt these to the given context. Future frameworks should aim to collaborate with PWD and consider the subjective components of accessibility [64].

## Supplementary Material

PRISMA_ScR_Checklist_Frameworks_to_analyse_accessibility_of_public_recreational_spaces_for_persons_with_disabilities_clean.docx

Appendix A.docx
