# Supplementary material for: Frameworks to analyse accessibility of public recreational spaces for persons with disabilities: a rapid scoping review
Source: Glob Health Action. 2026 Sep 14;19(1):2729891. doi: 10.1080/16549716.2026.2729891 (PMC13577271; doi:10.1080/16549716.2026.2729891)
Supplement: Supplementary file 1 [file ZGHA_A_2729891_SM2864.docx]

**Appendix A: Scoping review protocol**

**Scoping review Protocol: Frameworks and interventions to improve access to public recreational space for people with disabilities.**

Purpose: To examine and map the frameworks that have been used to analyse disability inclusion in recreational public spaces. This mapping will inform the analysis of data and development of locally relevant approaches relevant for ongoing research about how people with disabilities use outdoor fitness equipment and engage in physical activity. If no frameworks to analyse inclusion in recreational public spaces currently exist, then the scoping review will serve as a foundation for creating a new framework or combining/extending existing but less specific frameworks.

**Stage 1: Research question**:

1. What theoretical and conceptual frameworks currently exist to analyse disability inclusion in recreational public spaces?

**Stage 2: Parameters of review**

- Electronic databases: Scopus, Web of Science, ASSIA, Jstor, Embase Google Scholar, and Google
- Given the available time these searches are the main priority – this also means only searching the first 10 pages of google pages
- Reference lists of papers
- Existing networks (e.g. DPOs and organisations for people with disabilities in Nepal)
- Relevant organizations and conferences (e.g. UNICEF, IDA, HI, CBM, UNHCR)

Search terms to use:

Public space AND accessibility AND Framework

Public space category

(Public space or park or recreation space or playground or gym or outdoor gym or fitness space or outdoor exercise equipment)

Accessibility category

(Accessibility or disability or adaptive or inclusion or physical impairment or mental impairment)

Framework category

(Framework or schema or method of analysis or system of analysis)

Definitions

- Public space – “open space accessible to all”[1]
- Recreation – “activities that people choose to do to refresh their bodies and minds and make their leisure time more interesting and enjoyable”[2]
- Disability –the social, structural, and environmental factors which fail to account for physical or mental impairments of people[3]
- Intervention (in the context of public health) – “an organized set of means implemented in a specific context to meet one or several targets with respect to improving health and preventing disease”[4]
- Framework – “denotes a structure, overview, outline, system or plan consisting of various descriptive categories, e.g. concepts, constructs or variables, and the relations between them that are presumed to account for a phenomenon”[5]

**Stage 3: Inclusion and exclusion criteria**

Include: papers from the last 20 years (2005-) in English, papers from LMICs and HIC, papers that propose or apply a framework of analysis to assess the inclusivity of public recreational spaces.

Exclude: papers focusing on organized play/sport, papers not including disability/impairment, papers only focusing on children, papers specific to private recreational spaces

**Stage 4: charting the data**

The papers will be charted in an excel file, collecting relevant information from included papers.

**Stage 5: write up**

The findings from the scoping review will be used in a manuscript about ongoing work.

**Stage 6: Consultation**

We will discuss the protocol and our findings with a person with disabilities and with our collaborator who is a person with disabilities.

References

1.	Parashar A, Lakra HS. Studying public spaces – a Systematic literature review. Journal of Urbanism: International Research on Placemaking and Urban Sustainability. 2023:1-32. doi: 10.1080/17549175.2023.2280631.

2.	Khasnabis C HMK, Achu K, et al. Community-Based Rehabilitation: CBR Guidelines. Geneva: World Health Organization; 2010.

3.	Haegele JA, Hodge S. Disability discourse: Overview and critiques of the medical and social models. Quest. 2016;68(2):193-206.

4.	Litvak E, Dufour R, Leblanc É, Kaiser D, Mercure S-A, Nguyen CT, et al. Making sense of what exactly public health does: a typology of public health interventions. Canadian Journal of Public Health. 2020;111(1):65-71. doi: 10.17269/s41997-019-00268-3.

5.	Nilsen P. Making sense of implementation theories, models and frameworks. Implementation Science. 2015;10(1). doi: 10.1186/s13012-015-0242-0.
